# Oral health behavior of drug addicts in withdrawal treatment

**DOI:** 10.1186/1472-6831-13-11

**Published:** 2013-01-31

**Authors:** Hajar Shekarchizadeh, Mohammad R Khami, Simin Z Mohebbi, Jorma I Virtanen

**Affiliations:** 1 Community Oral Health Department, School of Dentistry, Tehran University of Medical Sciences, North Kargar Street, Tehran 1439955991, Iran; 2 Department of Oral Public Health, Institute of Dentistry, University of Helsinki, P.O.Box 41, Helsinki FI-00014, Finland; 3 Department of Community Dentistry, University of Oulu, P.O.Box 5281, Oulu, FI-90014, Finland; 4 Department of Public Health, University of Helsinki, P.O.Box 41, Helsinki, FI-00014, Finland

**Keywords:** Addiction, Substance abuse, Withdrawal treatment, Methadone maintenance, Oral health behavior

## Abstract

**Background:**

Oral health behavior (OHB), one major factor contributing to proper oral health status, has been addressed insufficiently in addiction literature. The aim of our study was to investigate OHB and its determinants among drug addicts in withdrawal treatment.

**Methods:**

Through a stratified cluster sampling method, we collected the data from 685 patients in withdrawal treatment in Tehran using self-administered questionnaires on OHB components and conducting interviews about patients’ characteristics and addiction history. The T-test, ANOVA, and a linear regression model served for statistical analysis.

**Results:**

Of the patients, 48% reported brushing their teeth less than once a day, more than 90% used fluoride toothpaste almost or always, and 81% flossed their teeth rarely or never. Eating sugary products twice a day or more was reported by 57% of the patients and 85% of them were current smokers. Poor OHB was associated with male gender, lower education, being addicted mainly to crystalline heroin, starting drug abuse at a younger age, and having a longer history of addiction (*p* < .05).

**Conclusion:**

Poor OHB was found among the participants in drug withdrawal treatment. Preventive strategies on oral health should be planned and be integrated into other health promotion programs for addicts along with their withdrawal treatment taking into account special groups at higher risk.

## Background

Drug abuse results in various individual and social consequences and takes a heavy toll in terms of severe health complications, risky behaviors, violence, and social problems [1]. In addition to the direct effects of drugs on oral health, drug abuse may aggravate oral problems indirectly through its adverse effects on user’s behavior and life style. Poor oral hygiene, a tendency toward consumption of sweet foods, irregular eating patterns, poor nutrition, and irregular dental visits are prevalent among addicts [2,3]. In fact, for addicts, the effects of social and behavioral factors on their oral diseases are greater than is the effect solely of drugs. Factors such as socio-demographic background, childhood access to dental care, sugar consumption, and poor oral hygiene, as well as smoking, seem to influence the oral health complications among addicts [4].

Because oral health is an important part of general health, health and wellbeing are likely to be improved by all efforts at oral health promotion. In oral health promotion activities, as well as in health promotion programs, the setting plays an important role. This has led many people working in this field to adopt a setting approach [5]. Rehabilitation settings such as addiction treatment centers help patients feel safe and help them take control of their lives and focus more on their health [6,7]. These settings hence can play an important role in changing patients’ behavior [8]. These centers can also be considered as a great opportunity for programs targeting oral health behavior (OHB) to become integrated into other services provided for addicts. Improved access to dental health care via these settings might lead to changing the patient’s behavior and life style and, in turn, to help improve the success of withdrawal treatment [9]. In order to plan suitable and successful oral health-promotion programs, the existing situation of patients’ oral health behaviors have to be assessed.

While OHB is one of the major contributing factors to proper oral health status, not much is known about the addicts’ behaviors. Few available reports from the UK and USA highlight the poor OHB among these patients in terms of brushing habits, snacking, and recent visits to a dentist [2,3,6]. Although Iran endures high rates of addiction to opiate drugs [10] and about 1.2 million addicts in need of treatment live in the country [11], no comprehensive published data exist on this group’s OHB. In total, 700 private and 150 public centers, 160 of which are located in Tehran, offer outpatient services to patients [11].

The aim of our study was to investigate the OHB of drug addicts in withdrawal treatment. In addition, we analyzed the associations between OHB and users’ characteristics and addiction history in order to identify special subgroups at greater risk.

## Methods

### Subjects

The present study was performed in addiction treatment centers in Tehran, Iran. The target population comprised drug users receiving methadone maintenance treatment (MMT) at these centers from January to May 2011. A multi-stage stratified cluster random sampling technique covered the three main socioeconomic areas of the city: north, center, and south. After dividing the city into these three main strata, we selected clinics at random as clusters. Since more than 95% of treatment centers were private clinics, in order to achieve a homogenous group, we excluded public centers [12].

Considering the proportion of clinics in each of the three strata, we randomly selected the clinics as clusters in each stratum. In total, eight clinics (3 of 59 in the south, 3 of 64 in the center, and 2 of 27 in the north of the city) entered the survey. To estimate sample size, proportional sampling was applied and sample size of at least 320 persons was calculated. Because of the high number of edentates among addicts and to be able to compare various subgroups we extended data collection to cover the monthly turnover of MMT patients in the selected clinics. Of 813 participants (response rate: 72%), 128 (16%) were totally edentulous and were excluded from the study.

### Data collection

The data were collected with both self-administered questionnaires and with conducting interviews. Three trained senior dental students conducted the survey. Before data collection, the investigators underwent training about the content of the interview and the questionnaire, and the method of communication with the patients. A detailed description of data collection appears in an earlier report [12].

#### Self-administered questionnaire and interview

In the clinics, after a brief explanation of the study, the investigators asked the patients who volunteered to fill in the questionnaire on oral health. Completing the questionnaire took around 20 minutes. Participants were able to ask for explanations of the questions if necessary. The questionnaire requested information on OHB components based on previously validated questionnaires [13,14]. The questionnaire and the feasibility of the method were pretested in a pilot study prior to data collection.

To obtain patients’ addiction history and background factors, the investigators conducted 10-minute structured in-person interviews by use of a standard patient-characteristic form commonly serving as a framework in addiction studies in Iran [12]. Based on the monthly turnover of patients in each clinic, we continued data collection in the clinics to cover all eligible patients.

##### Oral health behavior

Oral self-care comprised questions about the frequency of tooth brushing, using fluoride toothpaste, flossing, eating sugary products between the main meals, and smoking. Dental attendance comprised a question as to the time of the most recent visit to a dentist [13-16]. Each question had between 4 and 7 alternatives. To obtain a total score for OHB for each patient, based on the authors’ experience and the relevant literature [17-19], appropriate weights were assigned to all components, with higher weights for health-promoting behaviors: frequency of tooth brushing (0 = less than once daily, 2 = once daily, 4 = twice daily or more), using fluoride toothpaste (0 = rarely or never, 1 = almost or always), flossing (0 = never or sometimes, 1 = several times weekly, 2 = at least once daily), eating sugary products between meals (0 = twice daily or more, 2 = once daily, 4 = not daily or rarely), smoking (0 = daily smoking, 1 = current but not daily smoking, 2 = no current smoking), and time of most recent visit to a dentist (0 = never or more than 2 years ago, 1 = between one and two years ago, 2 = during the previous year). We then summed up the scores for the components to calculate the total OHB score with a range from 0 to 15.

##### Addiction history

We asked the patients about their main drug of abuse (the most problematic drug which made the patient enter treatment), age when drug abuse began, duration of addiction, and duration of current treatment.

##### Background factors

We asked the patients their gender and age*.* In addition, their residential area, education, and marital and employment status served as indicators for socioeconomic status (SES). These variables were dichotomized as follows: for education, higher education (diploma or higher) = 1; lower education (less than diploma) = 0; for marital status, married = 1; single (single, widow, divorced) = 0; for employment status, employed (full or part-time) = 1; unemployed (unemployed, retired, homemaker, and student) = 0; and for residential area, affluent (north and center) = 1; non-affluent (south) = 0. These were combined by summing up the dichotomized scores (range: 0 to 4).

### Ethical approval

The Tehran University of Medical Sciences Ethics Committee approved the study. Participation in the study was voluntary, and all respondents provided their written informed consent. To provide as much confidentiality as possible, we used an anonymous patient-characteristic form and an anonymous questionnaire for data collection.

### Statistical analysis

The data was analyzed with the Statistical Package for the Social Science (SPSS for Windows, version 18.0/PC; SPSS, Chicago, IL, USA). Student’s t-test and analysis of variance (ANOVA) served for statistical analysis (level of significance < .05). A linear regression model was fitted to the data to analyze the relationship between independent variables and total OHB score.

## Results

Among 685 dentate patients who completed the questionnaire, 3 did not agree to be interviewed resulting to a sample size of 682 participants. The mean age of the patients was 38.2 years (SD 10.1; range 20–79); 96% were men. Their background characteristics and addiction history are presented in Table 1. The main drugs of abuse were opium (65%) and crystalline heroin (27%), and the age when drug abuse began was 18 to 24 years for almost half of the patients.

**Table 1:** Characteristics of addicts in withdrawal treatment (n = 682)

**Variable**	**Number**	**Percentage**
**Gender**		
Male	656	96
Female	29	4
Total	685^a^	100
**Age**		
18-24	30	4
25-34	268	39
35-44	214	32
45-54	119	17
55 ≤	51	8
Total	682	100
**Education status**		
0-5	64	10
6-12	436	65
13 ≤	168	25
Total	668	100
**Employment status**		
Full-time job	385	57
Part-time job	133	19
Unemployed	101	15
Others (student, retired, homemaker)	62	9
Total	681	100
**Main drug of abuse**		
Opium	444	65
Crystalline heroin	183	27
Others	52	8
Total	679	100
**Age of starting drug abuse (years)**		
≤ 17	103	15
18-24	311	46
25-34	190	28
35 ≤	73	11
Total	677	100
**Duration of addiction (years)**		
< 1	21	3
1-5	210	31
6-10	205	31
11 ≤	236	35
Total	672	100
**Duration of current treatment (months)**		
< 1	79	12
1-5.9	193	28
6-11.9	149	22
12-23.9	124	18
24 ≤	133	20
Total	678	100

^a^ Regardless of interview, participants’ gender was recognized based on their appearance.

Table 2 presents the OHB of the drug users in withdrawal treatment. Around half the patients (48%) reported brushing their teeth less than once daily. More than 90% used fluoride toothpaste almost or almost always. A clear majority (81%) never or rarely flossed their teeth. More than half the patients (57%) reported eating sugary products twice daily or more often between their main meals. Of the patients, 85% were current smokers, among whom a great majority were daily smokers. Although more than half the patients (57%) had visited a dentist during the previous year, for 25% of the participants the most recent visit was more than 2 years previously or never.

**Table 2:** Oral health behavior profile of addicts in withdrawal treatment (n = 685)

**Variable**	**Alternatives**	**Frequency (%)**
**Tooth brushing**	Not every day	324 (48)
	Once daily	266 (39)
	Twice daily or more	85 (13)
	Total	675
**Use of fluoride toothpaste**	Rarely or never	47 (7)
	Almost or always	625 (93)
	Total	672
**Dental flossing**	Never or sometimes	551 (81)
	Several times a week	33 (5)
	Once daily or more	93 (14)
	Total	677
**Consuming sugary snacks**	Twice daily or more	385 (57)
	Once daily	111 (16)
	Not every day or rarely	182 (27)
	Total	678
**Smoking**	Daily smoking	521 (78)
	Current but not daily smoking	45 (7)
	No current smoking	105 (15)
	Total	671
**Time of most recent visit to a dentist**	More than 2 years ago or never	158 (25)
	1-2 years ago	115 (18)
	Previous year	362 (57)
	Total	635

The mean OHB score was 5.6 (SD 3; range 0–15) among participants. Table 3 presents the mean OHB scores according to patients’ characteristics and addiction history. In univariate analysis, a higher OHB score was significantly associated with female gender, older age, and higher education (*p* < .05). Moreover, patients categorized as “other” regarding employment status (student, retired, homemaker), and those from clinics located in the center region had higher OHB scores (*p* < .001). A significant association existed between OHB score and patients’ addiction history: crystalline heroin users had lower OHB scores (*p* = .007). Patients who had started using drugs at a younger age and those who had a longer history of addiction showed significantly lower OHB scores (*p* < .001). Duration of current treatment was not associated with patients’ OHB.

**Table 3:** Oral health behavior (OHB) scores of addicts according to their backgrounds and addiction history (n = 682)

**Variable**	**OHB score (Mean, SD)**	***p*-value**
**Gender**		
Male	5.6 (2.9)	.020*
Female	7.0 (3.3)	
**Age**		
18-24	5.3 (2.4)	<.001**
25-34	5.5 (2.8)	
35-44	5.4 (3.1)	
45-54	5.6 (2.8)	
55 ≤	7.8 (3.2)	
**Education**		
0-5	4.3 (2.9)	<.001**
6-12	5.4 (2.7)	
13 ≤	6.9 (3.1)	
**Marital status**		
Married	5.7 (3.0)	.050**
Single	5.8 (3.0)	
Others	4.7 (2.6)	
**Employment status**		
Full-time job	5.6 (3.0)	<.001**
Part-time job	5.3 (2.8)	
Unemployed	5.1 (2.8)	
Others (student, retired, homemaker)	7.5 (2.8)	
**Area of residence**		
North	5.6 (3.1)	<.001**
Center	6.4 (2.9)	
South	5.0 (2.8)	
**Main drug of abuse**		
Opium	5.8 (3.0)	.007**
Crystalline heroin	5.0 (2.6)	
Other drugs	6.3 (3.4)	
**Age of starting drug abuse**		
≤ 17	4.9 (2.6)	<.001**
18-24	5.5 (2.8)	
25-34	5.7 (3.1)	
35 ≤	7.2 (3.3)	
**Duration of addiction (year)**		
< 1	7.9 (2.8)	<.001**
1-5	6.4 (3.0)	
6-10	5.2 (2.9)	
11 ≤	5.1 (2.8)	
**Duration of current treatment (month)**		
< 1	5.8 (2.9)	.563**
1-5.9	5.5 (2.9)	
6-11.9	5.4 (3.0)	
12-23.9	6.0 (3.2)	
24 ≤	5.7 (2.8)	

* t-test, **ANOVA.

The linear regression model (Table 4) confirmed the results of univariate analysis: having been addicted to crystalline heroin as one’s main drug, starting drug abuse at a younger age, and longer duration of addiction were associated with lower OHB scores (*p* < .05). In addition, female gender and higher SES were significantly associated with higher OHB scores (*p* < .05).

**Table 4:** Factors associated with oral health behavior scores of addicts by a linear regression model

**Model**	**Unstandardized coefficients**		**Standardized coefficients**	***p*-value**	**95% Confidence interval for B**	
	**B**	**Standard error**	**Beta**		**Lower bound**	**Upper bound**
**Gender**^a^	2.15	0.62	0.14	.001	0.93	3.37
**Socioeconomic status**^**b**^	0.43	0.12	0.14	<.001	0.19	0.66
**Main drug of abuse**^c^	0.60	0.27	0.09	.027	0.07	1.14
**Age of starting drug abuse**	0.07	0.02	0.17	<.001	0.04	0.10
**Duration of addiction**	−0.05	0.02	−0.11	.008	−0.08	−0.01

^a^ male = 0, female = 1.

^b^ Sum variable of the following variables: education, (diploma or higher = 1; less than diploma = 0); marital status, (married = 1; single = 0); employment status, (employed = 1; unemployed = 0); and residential area, (affluent =1; non-affluent = 0).

^c^ crystalline heroin = 0, other drugs = 1.

R^2^ = 0.11.

## Discussion

One of the prevalent problems among drug users is oral health diseases requiring serious attention, since a mutual relationship seems to exist between addiction and oral complications: addiction can cause oral diseases [20-23], but then oral health problems may also lead to intake of illicit drugs for pain relief [24]. The topic of oral health, however, has not been addressed sufficiently in addiction literature [25]. The present study of oral health behavior and its determinants reveals generally poor OHB by these patients in withdrawal treatment. For crystalline heroin users especially, those who started drug abuse at younger ages and had been addicted longer had inadequate OHB.

### Oral self-care profile of addicts

The present study revealed poor oral self-care among addicts receiving MMT, most of them not meeting the criteria for the recommended level of oral self-care components: tooth brushing at least twice daily, application of fluoride toothpaste always or almost always, and snacking on sugary products between meals less than once daily [15]. As only about 14% of the patients brushed at least twice daily and flossed on a daily basis, 73% snacked on sugary products once daily or more often and 85% were current smokers. In a study of adult methamphetamine users in Iowa, 6% of the patients reported brushing twice daily or more often, and 17% used dental floss daily [2]; these findings are comparable to ours. In-treatment drug users in Delhi have reported similar findings on frequency of cleaning the teeth (4%) [26]. In addition, Brazilian addicts undergoing rehabilitation showed a similar habit of eating between meals as in our study; however, dental flossing was reported by as many as 30% of those patients [27]. Another study involving Italian alcohol-addicted patients in residential rehabilitation clinics showed that the frequency of twice daily or more frequent brushing was 54% [8], a much higher figure than we found. This emphasizes an urgent need to develop efficient oral self-care instructions for such vulnerable individuals.

The literature on OHB of addicts is scarce. However, in a comparable special-needs patient group of hospitalized Danish psychiatric patients, tooth brushing at least twice daily was practiced by 55% of the patients [28]. In another study regarding oral self-care among diabetic patients in Sweden, tooth brushing at least once a day was reported by 91% and proximal cleaning by 52% of the patients [19]. In Iran, a study of OHB among a group of diabetic adults reported higher levels of recommended OHB than in our study: brushing (28%), and flossing (47%) [29]. These comparisons point out the overall poor OHB among addicts and call attention to the serious need for improving their behavior via preventive oral health care instructions. Given the fact that rehabilitation settings play an important role in behavioral change [8], oral health promotion programs should be integrated into other services provided for addicts in treatment centers in order to achieve stable treatment outcomes.

### Dental attendance of addicts

Regarding use of dental services, around half the participants in our study (43%) had not visited a dentist during the previous year. Similar findings have emerged among drug users in the USA, with around 52% of drug users having their most recent dental visit more than one year ago [30], and in the UK, with addicts (54%) less likely to visit a dentist during the previous year than were non-drug users (84%) [3]. Dental attendance at an even lower rate occurs among alcohol-addicted patients in Italy (37%) [8], and for example in psychiatric patients in Denmark (31%) [28].

Dental attendance among MMT patients in our study was comparable to that of the general adult population in Tehran (52%) [31] and also to that of Iranian diabetic patients (47%) [29]. This implies that unlike in the UK [3], underlying factors associated with dental attendance in Iran may be rather similar among various groups. However, such factors as problems in registering with a dentist, having been refused access to treatment, stigmatization, and negative attitudes of health professionals toward drug users, users’ anxiety and fear of dentists, low importance to them of personal appearance, and poor socioeconomic status may be associated with dental visits among drug users [3].

### Factors associated with OHB of addicts in withdrawal treatment

OHB among addicts was clearly associated with background and socioeconomic status. Poor OHB was not equally distributed among the MMT patients: male participants and those less well educated were at greater risk. This is in line with other studies of OHB in general populations and among certain groups [18,28,32-34]. However, since the gender distribution in the study was skewed, this result should be interpreted with caution.The lowest score of OHB was among unemployed participants, with a higher level of OHB for students, retired people, and homemakers, which might be due to the latter two groups’ having more free time, and because of the importance of good appearance among the students [35]. Participants from clinics located in the central region reported better OHB, whereas the lowest OHB score existed among patients from the southern region which reportedly had the lowest level of SES, in line with findings for Slovenia [18].

It is noteworthy that inadequate OHB appeared especially among crystalline heroin users, those who reported starting drug abuse at a younger age, and ones with longer history of drug abuse. Thus, it seems that not only addiction but also the kind of drug used and the characteristics of drug abuse may play an important role in OHB [22]. These findings may have implications for oral health promotion among addicts through targeting of specific subgroups. General practitioners and specialists in substance abuse should advocate oral health care and integrate it into other general care in treatment settings and educational programs.

### Strengths and limitations of the study

The stratified cluster random sampling method and the large sample size of 813 with a high response rate of 72% provided a good overview of the patients in treatment. The study focuses on the extent and distribution of oral health-related behaviors of drug addicts in withdrawal treatment in Tehran and thus casts light on a field not researched in developing countries. The present study was cross-sectional in nature. Since this population is not readily accessible, a representative sample of the whole population of addicts is difficult to achieve, meaning that we cannot generalize these findings to all drug users [12]. Our subjective data collection (self-reported data) may be another limitation of the study. We tried to overcome the possibility of over-reporting of desirable behaviors by using an anonymous self-administered questionnaire to obtain an accurate picture of the situation.

## Conclusion

Our study demonstrates poor oral health behavior among addicts in withdrawal treatment, and especially those less educated and those addicted to crystalline heroin as being at greatest risk for oral diseases. Educational and preventive strategies on oral health should be integrated into other care provided for addicts, taking into account distinct patient subgroups.

## Abbreviations

OHB: Oral Health Behavior; MMT: Methadone Maintenance Treatment; SES: Socioeconomic Status.

## Competing interests

The authors declare that they have no competing interest.

## Authors’ contributions

All authors were involved in the conception and design of the study. H.S. carried out data collection; H.S. and J.V. performed statistical analyses and interpretation of the data. All authors participated in either drafting or critical revising the manuscript. The Final draft of the manuscript was approved by all authors.

## Pre-publication history

The pre-publication history for this paper can be accessed here:

http://www.biomedcentral.com/1472-6831/13/11/prepub
